# Diagnosis of amphimeriasis by LAMPhimerus assay in human stool samples long-term storage onto filter paper

**DOI:** 10.1371/journal.pone.0192637

**Published:** 2018-02-14

**Authors:** William Cevallos, Pedro Fernández-Soto, Manuel Calvopiña, María Buendía-Sánchez, Julio López-Abán, Belén Vicente, Antonio Muro

**Affiliations:** 1 Centro de Biomedicina, Carrera de Medicina, Universidad Central del Ecuador, Quito, Ecuador; 2 Infectious and Tropical Diseases Research Group (e-INTRO), Biomedical Research Institute of Salamanca-Research Centre for Tropical Diseases at the University of Salamanca (IBSAL-CIETUS), Faculty of Pharmacy, University of Salamanca, Salamanca, Spain; 3 Carrera de Medicina, Universidad De Las Américas (UDLA), Quito, Ecuador; Universidade Nova de Lisboa Instituto de Higiene e Medicina Tropical, PORTUGAL

## Abstract

Amphimeriasis, a fish-borne zoonotic disease caused by the liver fluke *Amphimerus* spp., has recently been reported as an emerging disease affecting an indigenous Ameridian group, the Chachi, living in Ecuador. The only method for diagnosing amphimeriasis was the microscopic detection of eggs from the parasite in patients' stool samples with very low sensitivity. Our group developed an ELISA technique for detection of anti-*Amphimerus* IgG in human sera and a molecular method based on LAMP technology (named LAMPhimerus) for specific and sensitive parasite DNA detection. The LAMPhimerus method showed to be much more sensitive than classical parasitological methods for amphimeriasis diagnosis using human stool samples for analysis. The objective of this work is to demonstrate the feasibility of using dried stool samples on filter paper as source of DNA in combination with the effectiveness of our previously designed LAMPhimerus assay for successfully *Amphimerus* sp. detection in clinical stool samples. A total of 102 untreated and undiluted stool samples collected from Chachi population were spread as thin layer onto common filter paper for easily transportation to our laboratory and stored at room temperature for one year until DNA extraction. When LAMPhimerus method was applied for *Amphimerus* sp. DNA detection, a higher number of positive results was detected (61/102; 59.80%) in comparison to parasitological methods (38/102; 37.25%), including 28/61 (45.90%) microscopy-confirmed *Amphimerus* sp. infections. The diagnostic parameters for the sensitivity and specificity werecalculated for our LAMPhimerus assay, which were 79.17% and 65.98%, respectively. We demonstrate, for the first time, that common filter paper is useful for easy collection and long-term storage of human stool samples for later DNA extraction and molecular analysis of human-parasitic trematode eggs. This simple, economic and easily handling method combined with the specific and sensible LAMPhimerus assay has the potential to beused as an effective molecular large-scale screening test for amphimeriasis-endemic areas.

## Introduction

Amphimeriasis, a fish-borne zoonotic disease caused by the liver fluke *Amphimerus* spp. (within the family Opisthorchiidae), was recently reported as an endemic disease in the tropical Pacific side of Ecuador. Data showing high prevalence of infection among an indigenous group, the Chachis, and also domestic cats and dogs residing in the same communities have been noted and, actually, human amphimeriasis has been reported as a new emerging food-borne zoonotic disease. Parasites of the genus *Amphimerus* infect humans after ingestion of raw or undercooked freshwater fish containing viable metacercariae. Human disease is mostly asymptomatic, occasionally causing non-specific, generalised symptoms. However, histopathological studies in cats and a double-crested cormorant infected with *Amphimerus* spp. showed the presence of liver cirrhosis and pancreatitis [1,2]. Similarly, as occur in other human infections by parasites of the family Opisthorchiidae, affected individuals with *Amphimerus* spp. can suffer from suppurative cholangitis, cholelithiasis and cholangiocarcinoma [3–5]. Since the Chachi community habitually consumes smoked freshwater fish, an estimated 13% of the inhabitants living along the Rio Cayapas in the Province of Esmeraldas are a risk of acquiring amphimeriasis [6].

Until very recently, the only method for diagnosing the disease was the microscopic detection of eggs from the parasite in patients’ stool samples, but it lacks in sensitivity [6]. To overcome this limitation, our investigation group developed, for the first time, an ELISA technique for detection of anti-*Amphimerus* IgG in human sera [7] and, afterwards, the first molecular method based on LAMP technology (named LAMPhimerus) for specific and sensitive parasite DNA detection. The LAMPhimerus method showed to be much more sensitive than the classical parasitological methods for amphimeriasis diagnosis using human stool samples for analysis [8]. In that study, a number of human stool samples from Chachi communities were preserved in 80% ethanol solution for later DNA extraction to test by LAMP assay. It is known that collection of fresh stool samples for diagnostic purposes can be quite difficult in some population groups. Besides, the handling, management and storage of a large number of patients' stool samples can be very laborious in large-scale field trials in poor settings with minimal infrastructures. This fact is especially true for many tropical diseases since they are frequently in populations remote from sophisticated diagnostic facilities. Dried samples spots or smears collected onto filter paper provide a potentially useful and economic means of overcoming these drawbacks. The use of dried specimens -especially blood and sera samples- for the diagnosis and surveillance of infectious diseases has been recently reviewed [9]. In general, dried specimens perform with sensitivities and specificities very similar to gold standard sample types when using for DNA extraction and subsequent analysis by PCR-based molecular methods. However, a standardization methodology is still needed. For collection, preservation and easy handling of stool samples onto filter paper there are very few cases, and only including protozoa studies [10–13].

It should be very interesting to join the advantages of using filter paper for easy collection and preservation of human stool samples and the easy LAMP technology [14]. Considering a number of salient advantages of LAMP over most PCR-based molecular methods [15, 16], LAMP technology shows a potential use in clinical diagnosis and surveillance of infectious diseases, particularly under field conditions in developing countries for most tropical diseases [17, 18].

As mentioned above, we have recently developed a sensible and specific LAMP assay for the successful detection of *Amphimerus* sp. DNA in human stool samples from a Chachi community. Now, the objective of this work is to demonstrate the feasibility of using dried stool samples on filter paper as source of DNA in combination with the effectiveness of our previously designed LAMPhimerus assay for successfully *Amphimerus* sp. detection in clinical stool samples.

## Materials and methods

### Ethics statement

The study protocol was approved by the Ethics Committee of Universidad Central del Ecuador (License number: LEC IORG 0001932, FWA 2482, IRB 2483. COBI-AMPHI-0064-11) and the Ethics Committee of the University of Salamanca (protocol approval number 48531). Participants were given detailed explanations about the aims, procedures and possible benefits of the study. Written informed consent was obtained from all subjects prior to the collection of biological samples for parasitological and molecular evaluation. Parents or guardians of children who participated in the study provided written informed consent on the child's behalf. All samples were coded and treated anonymously. Procedures were performed in accordance with the ethical standards laid down in the Declaration of Helsinki as revised in 2013.

### Study area and population

The study was conducted during February 2016 in two indigenous Chachi villages (El Progreso and Estero Vicente) in the Canton Eloy Alfaro alongside the Cayapas River in the Esmeraldas province, located in the northwest coastal rainforest of Ecuador, 320 km from the capital Quito. The indigenous Chachi -living together with Afro-ecuadorian and mestizo populations- is the predominant autochthonous group in this area, representing 13% of the inhabitants in this region. In these Chachi communities high prevalence of human (15.5% to 34.1%) and local cats and dogs (71.4% and 38.7%, respectively) with *Amphimerus* spp. have been previously reported [6, 19]. They live in remote villages where the only way to reach them is by boat along the river. Sanitation facilities are lacking. The members are hunters who typically eat undercooked freshwater fish (mainly smoked fish) caught in the neighboring rivers and food sharing is usually common [6, 19]. The main economic activities are agriculture, fishing and exploitation of forest resources. The province of Esmeraldas, forms part of the tropical rainforest known as “Choco Biogeográfico del Pacífico” which covers a section of the coast of Ecuador, Colombia and Panamá. This area has been labeled as a biological hotspot, an area with an extraordinary concentration of animal species. More details on the region can be accessed elsewhere [20].

### Human stool sampling and parasitological tests

A total of 102 participants living in two indigenous Chachi communities were enrolled in the study, including 56 females (54.90%) and 46 males (45.09%) with a median age of 20.39 (range 1–65 years). Each participant was given a copro-parasitological flask for stool collection. Samples were collected within a few hours of stool passing. A single stool sample was individually obtained from each participant. After collection, samples were transported to the laboratory of parasitology (Centro de Biomedicina, Universidad Central del Ecuador, Quito, Ecuador) for parasitological screening under light microscopy by simple sedimentation technique (SST), formalin-ether concentration technique (FECT) and Kato-Katz technique (KKT). All samples were examined by two qualified laboratory technicians according to the basic laboratory procedures in Medical Parasitology, recommended by the World Health Organization (WHO) [21].

In addition, a portion of each sample was spread with a swab onto a filter paper (10 X 2 cm, approximately), air-dried, numbered, folded in a half, and individually wrapped in foil. In that way, samples were stored at room temperature until shipped to the Research Centre for Tropical Diseases at the University of Salamanca, Spain, for further DNA extraction (during February 2017) and molecular analysis as described below.

### DNA extraction for molecular analyses

#### DNA from parasites

*Amphimerus* sp. genomic DNA was extracted from frozen adult worms that were previously obtained from the livers of naturally infected cats and dogs of Chachi communities, as described elsewhere [19], using a G-spin Total DNA Extraction Kit (Intron Biotechnology) according to the manufacturers’ instructions. DNA was measured using a Nanodrop ND-100 spectrophotometer (Nanodrop Technologies) and then diluted with ultrapure distilled water to final concentration of 0.5 ng/μL to use as positive control in all LAMP reactions.

#### DNA from human stool samples smeared on filter papers

**Fig 1:**
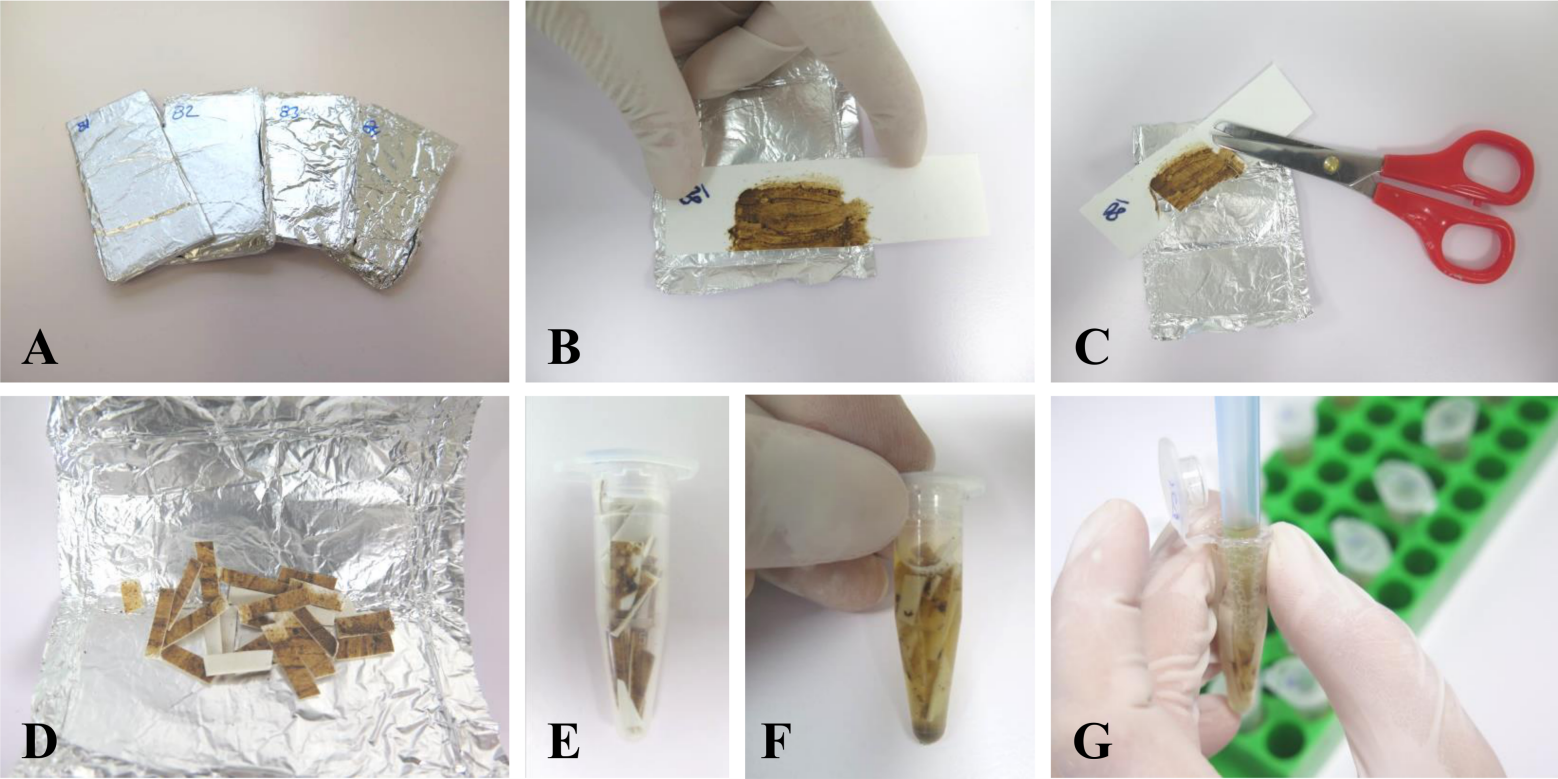
Human stool samples processing for DNA extraction. A. Batches organization. B. Smeared stool sample on filter paper. C, D. Filter paper is cut with scissors into thin strips. E, F Thin strips of each sample are placed into a 1.5 mL tube immersed in a lysis mixture an incubated for 30 min at 65°C in a thermoblock G. Mixture is transfer for begining DNA extraction with the commercial kit i-genomic Stool DNA Extraction Mini Kit (iNtRON Biotechnology).

DNA from human stool samples smeared on filter papers was extracted 12 months after collection and preparation. Steps followed for DNA extraction are shown in Fig 1. DNA extraction procedure was performed in batches of 10 samples each for easy handling and also to prevent potential cross-contamination. DNA extraction was performed using the i-genomic Stool DNA Extraction Mini Kit (Intron Biotechnology) according to the manufacturers' instructions with some additional procedures as follows. The smeared portion of filter papers were cut with scissors into thin strips. Scissors were always sterilized before cutting the next sample to prevent contamination. Thin strips of each sample were first placed into a 1.5 mL tube immersed in a lysis mixture -TE (400 μL; pH 8.0), lysis buffer (200 μL Buffer SL) and proteinase K (20 μL)-, vortexed vigorously, and subsequently incubated for 30 min at 65°C in a thermoblock. During incubation, to help dissolve feces until complete lysis the tubes were vortexed or inverted at about 5–10 min intervals. After incubation, a volume of 500 μL approximately of the mixture was transferred into a IR Spin Column for proper binding, washing, and elution steps. A final eluate of 100 μL of genomic DNA (gDNA) was obtained from each sample and divided into two aliquots of 50 μL each. After measuring the concentration using a Nanodrop ND-100 spectrophotometer (Nanodrop Technologies), DNA samples were stored at -20°C until use in molecular assays.

### LAMPhimerus assay

All the human stool samples were tested using the reaction mixture and specific primer set for LAMP assay (LAMPhimerus) previously established by our group [8]. The LAMPhimerus method amplifies a sequence of the *Amphimerus* sp. internal transcribed spacer 2 region (GenBank acc. no. AB678442.1). Briefly, the reaction was carried out with a total of 25 μL reaction mixture containing 40 pmol of each FIP and BIP primers, 5 pmol of each F3 and B3 primers, 1.4 mM of each dNTP (Intron), 1x Isothermal Amplification Buffer -20 mM Tris-HCl (pH 8.8), 50 mM KCl, 10 mM (NH_4_)_2_SO_4_, 2 mM MgSO_4_, 0.1% Tween20- (New England Biolabs, UK), 1 M betaine (Sigma, USA), supplementary 6 mM of MgSO_4_ (New England Biolabs, UK) and 8 U of *Bst* 2.0 WarmStart DNA polymerase (New England Biolabs, UK) with 2 μL of template DNA. Reaction tubes were placed in an economic heating block (K Dry-Bath) at a constant temperature of 63°C for 90–120 min and then heated at 80°C for 5 min to stop the reaction. In all LAMPhimerus trials positive controls (*Amphimerus* sp. gDNA) and a negative controls (water instead DNA) were included.

The LAMP amplification results could be visually inspected by the naked eye by colour change after adding 2 μL of 1:10 diluted 10,000X concentration SYBR^®^ Green I (Invitrogen) to the reaction tubes. To avoid as much as possible, the potential risk of cross-contamination with amplified products, all tubes were briefly centrifuged and carefully opened before adding the fluorescent dye. Green fluorescence was clearly observed in successful LAMP reaction, whereas it remained original orange in the negative reaction. The LAMP products (3–5 μL) were also monitored using 1.5–2% agarose gel electrophoresis, visualized under UV light and then photographed using an ultraviolet image system (Gel documentation system, UVItec, UK).

### Statistical analysis

To estimate the accuracy of the LAMP assay method as a diagnostic test, the percentages of the sensitivity, specificity, positive predictive value (PPV) and negative predictive value (NPV) were calculated using the MedCalc statistical program version 16.8.4 (MedCalc Software, Ostende, Belgium) according to the software instruction manual (http://www.medcalc.org).

## Results

### Parasitological tests

Of the total of 102 stool samples examined microscopically for the presence of *Amphimerus* eggs, 38 (37.25%) resulted positive at least by one of the parasitological techniques applied, including 27 (26.47%) positive by the simple sedimentation technique (SST), 19 (18.62%) positive by the formalin-ether concentration technique (FECT), and 27 (26.47%) positive by the Kato-Katz technique (KKT). Up to 15 (15/102; 14.70%) stool samples resulted simultaneously positive for the three parasitological tests; only 3 (3/102; 2.94%) stool samples resulted simultaneously positive for two parasitological tests, including 1 for SST and KKT and 2 for SST and FECT.

### LAMPhimerus analysis

**Fig 2:**
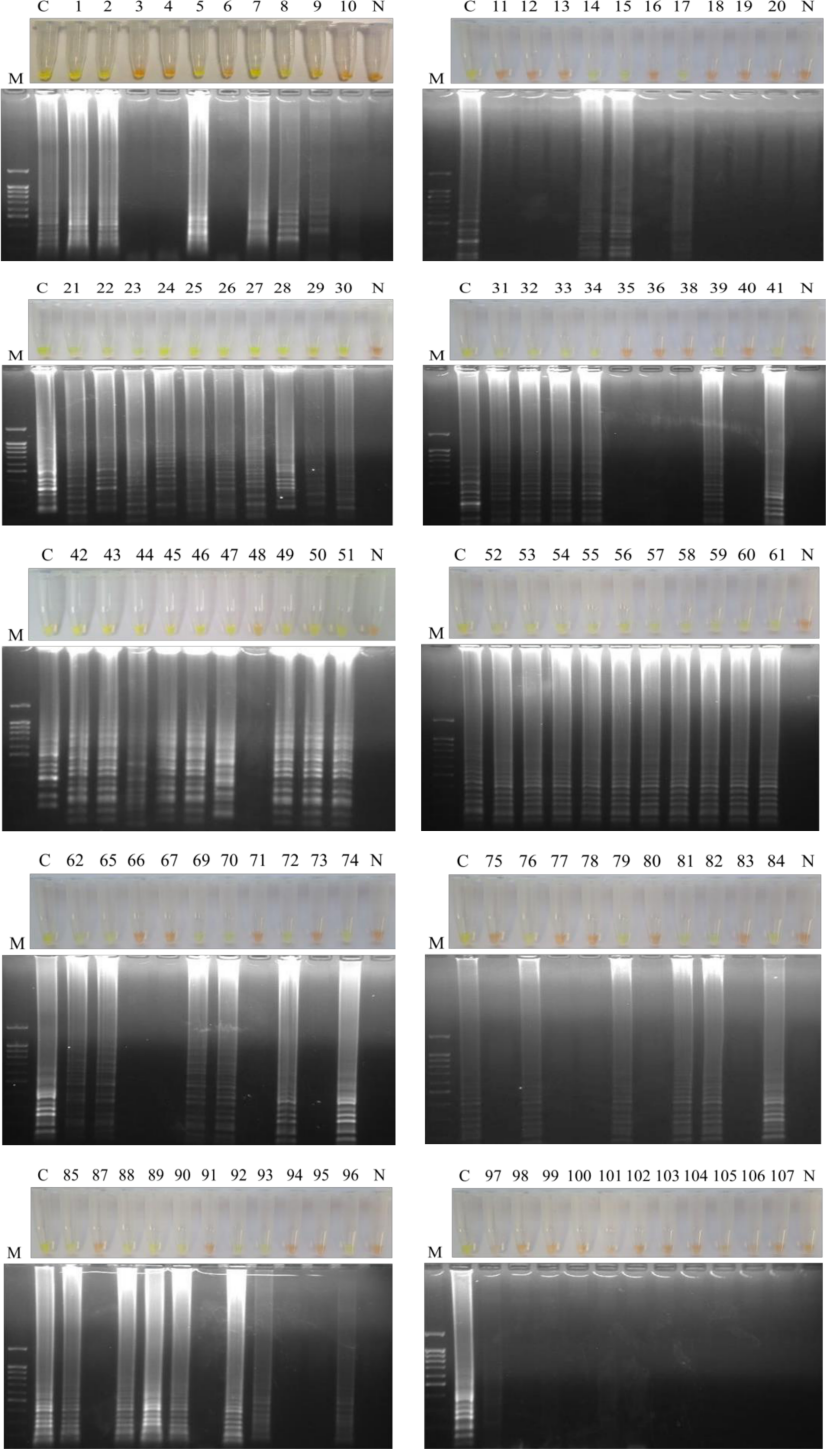
LAMPhimerus analysis of human stool samples in this study. Lanes M, molecular weight marker (100 bp Plus Blue DNA Ladder); lanes C, *Amphimerus* sp. genomic DNA (1 ng); lanes N, negative controls (ultrapure water and no DNA template); numbers 1–107, analyzed human stool samples.

Amplification assays were performed in batches of 10–11 samples each for easy handling and to prevent potential cross-contamination. All the samples were analyzed in duplicate with identical result. We obtained LAMP positive results in 61/102 (59.80%) samples, including 33/61 (54.09%) samples that were negative in all parasitological tests applied and 28/61 (45.90%) samples that were positive at least by one of the parasitological technique applied. Of the 15 samples (nos. 6, 27, 28, 30, 31 32, 33, 42, 54, 60, 79, 84, 85, 93, 97) that were simultaneously positive on three parasitological tests (FECT, SST and KKT), up to 13 (13/15; 86.66%) (nos. 27, 28, 30, 31 32, 33, 42, 54, 60, 79, 84, 85, 93) were also positive by LAMPhimerus assay; only 2 samples (2/15; 13.33%) (nos. 6 and 97) were negative on the LAMPhimerus assay. In all LAMP positive amplifications, green fluorescence was clearly visualized under natural light conditions and also by electrophoresis in agarose gels (Fig 2). Positive controls always worked well and negative controls were never amplified.

**Fig 3:**
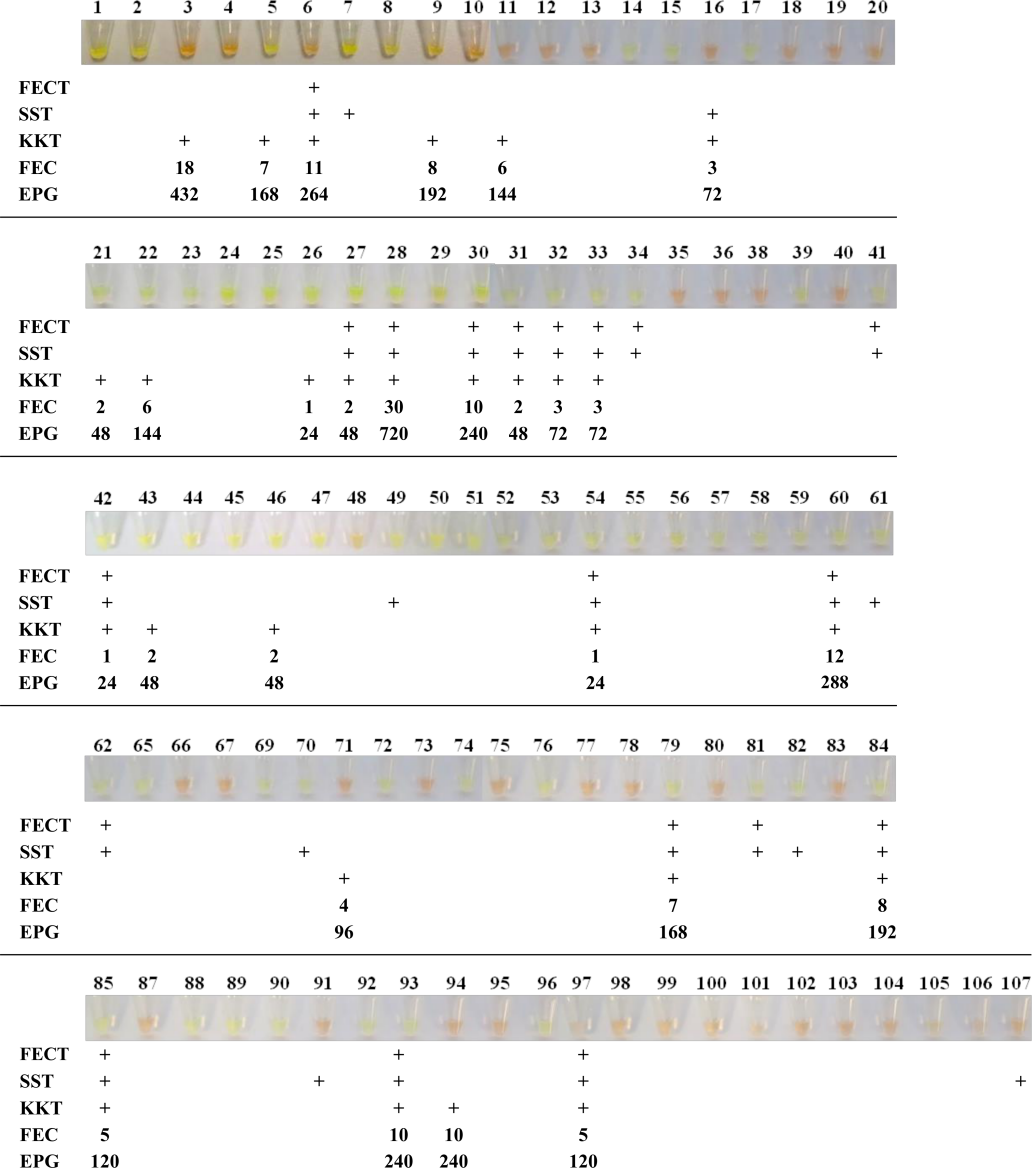
Comparison of the results obtained by the LAMPhimerus assay and classical parasitological techniques applied in this study. FECT, formalin-ether concentration technique; SST, simple sedimentation technique; KKT, Kato-Katz technique; FEC, fecal egg count; EPG, eggs per gram of feces; +, positive for egg detection. Values indicated for FEC and EPG correspond to the numbers of detected eggs. Numbers 1–107 correspond to the analyzed human stool samples.

In Fig 3 a total comparison of the results obtained by LAMPhimerus assay and parasitological techniques applied for detecting *Amphimerus* sp. in human stool samples is showed.

Considering the microscopy findings by parasitological techniques as the reference standard, the following diagnostic parameters for the sensitivity and specificity were calculated for our LAMPhimerus assay in this study: 79.17% sensitivity (95% CI: 65.0% -89.53%); 65.98% specificity (95% CI: 55.66% -75.30%); 53.52% positive predicted value (95% CI: 45.72% -61.16%) and 86.49% negative predicted value (95% CI: 78.36% -91.88%).

## Discussion

The indigenous Chachi communities, who live in remote villages along the Río Cayapas in the north-western coastal rainforest of Ecuador, have been shown to have a high prevalence of infection (15.5%-34.1%) with *Amphimerus* sp. [6]. Infection in domestic cats and dogs residing in this endemic area has also been reported as high (71.4% and 38.7%, respectively) and these animals have been proposed to serve as definite hosts and reservoirs for the parasite [19]. The prevalence data obtained in these studies were assessed according to eggs findings in both human and animal stool samples by classical parasitological methods.

Recently, in a pilot study using 44 human stool samples preserved in 80% ethanol solution from that area, a novel LAMP assay (LAMPhimerus) showed to be more sensitive than parasitological techniques for diagnosing human amphimeriasis [8]. Therefore, LAMPhimerus was proposed as a new molecular tool that could be readily adaptable for effective field diagnosis in amphimeriasis-endemic areas. However, the handling, management and storage of a large numbers of patients' fresh or frozen stool samples for diagnosing amphimeriasis in remote areas with poor infrastructure can be very difficult in large-scale field trials. Filter paper potentially provides a useful medium to overcome a number of difficulties of fresh sample collection, preservation and transportation. This method has been widely used as a specimen substrate when performing diagnostic or epidemiological surveys, especially in remote areas in resource-poor settings [9]. However, most studies have used filter papers for blood and sera collection and studies applying this method in human faecal samples for subsequent molecular detection of parasites are still very limited; a few reported examples are *Enterocytozoon bieneusi* [10, 11], *Giardia duodenalis* [12] and *Blastocystis* spp. [13]. Thus, the aim of this work is to demonstrate the feasibility of using filter paper for collection and preservation of human stool samples as source of DNA in combination with the effectiveness of our previously designed LAMPhimerus assay for successfully *Amphimerus* sp. detection in clinical stool samples.

There are several kinds and brands of filter paper available consisting of 100% cellulose and varying in thickness and pore size that have been used in different studies for PCR-based detection of DNA from humans, plants, animals, viruses, bacteria and parasites [9, 13]. In some cases, filter papers are impregnated with a proprietary mix of chemicals which provide protection of DNA of samples thus avoiding degradation and subsequent successful extraction. In addition, filter paper technology, such as FTA (Flinders Technology Associates)-treated matrix cards, may inactivate highly pathogenic organisms for safety transporting and long-term storage [13, 22]. However, some disadvantages of FTA paper are the use of a restricted diluted faecal sample volume of 15 μL for detection of protozoa and the whole procedure to get DNA template ready for PCR amplification takes approximately 3 hours [11].

In our preservation method, we used an economic common filter paper (100% cellulose with smooth surface and normal hardness) which is used for routine laboratory procedures such as basic filtration. Untreated and undiluted stool samples were spread as thin layer onto the filter papers for easily transportation to our laboratory and stored at room temperature for one year until DNA extraction. In our case, the whole procedure to get DNA template ready for LAMPhimerus assay, including the cutting of the strips, pre-incubation with the lysis mixture and DNA extraction with the commercial kit, can be performed in just 45 min. In addition, when measuring the DNA concentration of samples, the procedure yielded enough quantity of quality DNA for molecular detection by LAMPhimerus assay. According to this, long-term storage of dried stool samples onto common filter paper at room temperature worked very well for subsequent DNA extraction.

Thus, when LAMPhimerus method was applied to test human stool samples for *Amphimerus* sp. DNA detection, a higher number of positive results was detected (61/102; 59.80%) in comparison to parasitological methods (38/102; 37.25%), including 28/61 (45.90%) microscopy-confirmed *Amphimerus* sp. infection. It is important to note that up to 33/61 (54.09%) samples that were negative in all parasitological tests applied were LAMPhimerus-positive. These samples could be truly *Amphimerus* sp. infections undetected because of the known classically low sensitivity of the microscopy diagnosis in trematode infections [23]. This data reinforces the previous greater sensitivity of the LAMPhimerus assay over microscopic examination when testing human stool samples preserved in 80% ethanol solution [8]. On the other hand, only 8 truly parasitological *Amphimerus*-positive samples (nos. 3, 6, 11, 16, 71, 94, 97 and 107) were never amplified by LAMPhimerus assay. We think that the inoperative amplification in these samples was not due to the ineffectiveness of LAMPhimerus method because we obtained positive results in other microscopy-positive samples with lower EPG levels. Besides, the minimum amount of *Amphimerus* sp. genomic DNA detectable by LAMPhimerus assay (1 pg) has been reported to correspond to less than one single egg of the parasite in a stool sample [8]. An explanation for the inoperative amplification could be that the amount of the sample onto the filter paper was not enough to obtain *Amphimerus* sp. DNA for analysis. Perhaps, an inaccuracy in microscopy identification of parasite eggs occurred since morphological similarity of the *Amphimerus* spp. eggs to those of closely related species belonging to Opisthorchiidae family and to minute intestinal flukes makes diagnosis very difficult. Sometimes, it is necessary to use scanning electron microscopy to accurately observe the differences between the coatings on the different species [6]. This observation would further reinforce the specificity of LAMPhimerus method in the solely amplification of *Amphimerus* sp. DNA.

## Conclusions

In conclusion, to the best of our knowledge, we demonstrate for the first time that common filter paper is useful for long-term storage of human stool samples for later quality DNA extraction of human-parasitic trematode eggs. Additionally, this simple, economic and easily handling method combined with the specific and sensible LAMPhimerus assay has the potential to be used as an effective molecular large-scale screening test for amphimeriasis-endemic areas. The system 'air-dried stool sample on filter paper'-LAMP assay could also be very interesting and useful for molecular diagnosis of other human infectious parasitic diseases in remote areas with poor settings.

## Supporting information

S1 Checklist STARD checklist. (DOCX)
